# Bayesian Centroid Estimation for Motif Discovery

**DOI:** 10.1371/journal.pone.0080511

**Published:** 2013-12-06

**Authors:** Luis Carvalho

**Affiliations:** Department of Mathematics and Statistics, Boston University, Boston, Massachusetts, United States of America; Università degli Studi di Milano (University of Milan), Italy

## Abstract

Biological sequences may contain patterns that signal important biomolecular functions; a classical example is regulation of gene expression by transcription factors that bind to specific patterns in genomic promoter regions. In motif discovery we are given a set of sequences that share a common motif and aim to identify not only the motif composition, but also the binding sites in each sequence of the set. We propose a new centroid estimator that arises from a refined and meaningful loss function for binding site inference. We discuss the main advantages of centroid estimation for motif discovery, including computational convenience, and how its principled derivation offers further insights about the posterior distribution of binding site configurations. We also illustrate, using simulated and real datasets, that the centroid estimator can differ from the traditional maximum a posteriori or maximum likelihood estimators.

## Introduction

In motif discovery we are given a set of sequences that share a common motif and aim to identify the motif profile—the frequency of symbols for each position in the pattern—and the positions in each sequence where the motifs are. It is assumed that the motifs have significantly different profiles from sequence background. This problem has gained attention and relevance in the past 25 years mainly due to biological applications; a classical example is regulation of gene expression by transcription factors that bind to specific motifs in genomic promoter regions [1]–[3]. For this reason, we refer to the positions where the motifs are realized in the sequences as “binding sites”.

Due to its importance, hundreds of procedures have been proposed for motif discovery [4], [5]. While some approaches seek to characterize motifs and their binding sites using dictionary methods that capture over-representation of words as evidence [6], [7], it is common to represent motif compositions by a position weight matrix (PWM) [18] and specify a parametric model where sequences are generated conditionally on motif and background compositions and binding sites. Binding sites can then be regarded as missing data; parameters for the compositions can be estimated using expectation-maximization (EM) [9] in a frequentist setup [10], [11], or assigned a prior distribution in a Bayesian setup [12]–[14]. Other computational approaches are based on evolutionary algorithms and population clustering [15]–[17].

Even when exploiting prior information in both compositions and binding site configurations in a Bayesian setup, motif discovery is still considered a hard problem since motifs are usually short relative to sequence length and have a composition that might be hard to distinguish from background (see, for instance, [4].) It is then imperative to rely on more refined, informative estimation methods that better glean information from the posterior distribution of binding site configurations. Discrete inferential methods with this goal have recently been proposed, including the median probability model of Barbieri and Berger [8] and the centroid estimator [19], [20].

Estimators based on centroid inference, in particular, have been more successful than the ubiquitous maximum *a posteriori* (MAP) estimator when applied to motif discovery in models that account for sequence conservation [21], [22]. These estimators, however, were defined from a thresholded loss function that mostly compares binding sites across sequences instead of more finely comparing sequences position-wise for binding site overlaps, as in the traditional centroid estimator (details in Methods.) Moreover, these results rely on sampled binding site configurations to derive the centroid and thus do not offer a characterization of the estimator. Centroid estimators were also shown to yield more compact centered credibility sets then MAP estimators when applied to sequence alignment [23].

In this paper, we propose a novel centroid estimator that arises from a more refined and arguably more natural loss function and that can, in contrast to previous approaches, be fully characterized as a function of marginal posteriors in the space of binding site configurations. In this sense, we argue that the proposed estimator is a better representative of the posterior space of configurations. In addition, as a by-product of its derivation, we obtain informative summaries of the distribution of posterior mass. To this end, we adopt a Bayesian model for motif discovery on multiple sequences with multiple possible binding sites that is an extended version of the classic model from Liu, Neuwald, and Lawrence [14]. The motivation for this extended model is twofold: to obtain a feasible computational method while still retaining a realistic interpretation and to allow us to focus the discussion on the proposed estimator.

## Methods

We present our approach starting from a simple model and building up to the most general setup in the next sections.

### One Sequence, One Binding Site

Suppose we observe a sequence 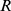, 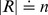, and wish to infer the location of the only binding site 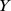, 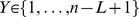. Following the Bayesian model from [14], we assume that there is only one motif of *fixed* length 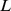 and that sequences are generated conditionally independently according to a product multinomial model given binding site positions and motif and background compositions. Thus, for an alphabet 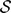, we define 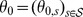 as background probabilities of generating each letter in 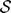 and, for each position 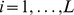 in the motif, 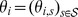 as the probabilities of generating each letter at the 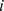-th position in the motif. To simplify the notation we denote 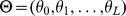. The likelihood is then:
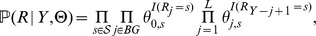
where 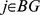 means position 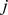 in background.

Setting a non-informative prior on 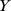, 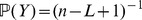, we have the posterior:
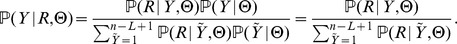
One traditional estimator is the maximum *a posteriori* (MAP) estimator,
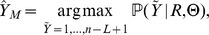
but we argue for an estimator that accounts for differences in positions when comparing binding site configurations. Using Bayesian decision theory [24] we look for an estimator that minimizes, on average, a more refined loss function 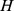:
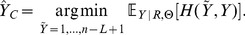
(1)We adopt a generalized Hamming loss 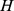,
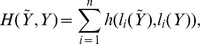
where 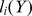 returns the “state” of position 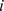: if 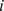 is a background position, 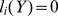, otherwise 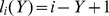, that is, 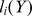 returns the position in the motif. Loss function 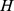 compares configurations position-wise according to 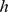, which in turn compares states. If we define 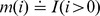 to indicate if state 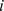 is a motif state then one option for 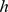 is 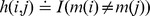, which yields a loss 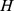 that accounts for overlap in binding sites. Such metric is commonly adopted to measure binding site level accuracy, as in the performance coefficients in [4], [5], [25].

Estimator 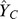 is a *generalized centroid estimator*; for instance, if 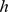 is a common zero-one loss, 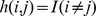, 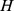 corresponds to Hamming loss, and thus 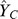 is the regular centroid estimator [19], [20]. As Carvalho and Lawrence [26] argue, centroid estimators more effectively represent the space since they are closer to posterior means; in contrast, it can be shown that 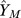 arises from a zero-one loss function which yields the posterior mode [26].

Let us now derive more specific expressions for 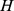 and 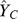. We first notice that if 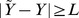 then the binding sites do not overlap and so 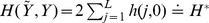, the null overlap distance between two configurations. Alternatively, when 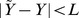 then
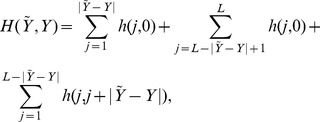
(2)since the common backgrounds in 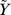 and 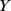 do not affect 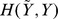, the first two terms above account for the left and right “tails” where binding sites in one sequence are matched with background in the other sequence, and the last term accounts for the overlap in binding sites. We also note that 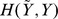 is actually a function of 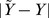.

Instead of a loss function we can also define our estimator in terms of a *gain* function 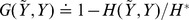. Note that 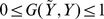; in particular, when 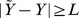 there is no gain, 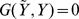, and if 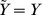 we have 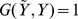. As a consequence, we can simply write 
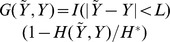
 with 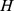 from Equation 2. Noting that 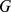, like 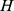, is also a function of 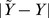, we obtain the following characterization:

**Theorem 1** *The centroid estimator* 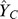 *is*

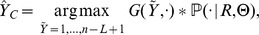
a *convolution between* 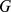 *and the posterior distribution on* 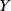.

*Proof.* The result follows directly from the definition in Equation 1:
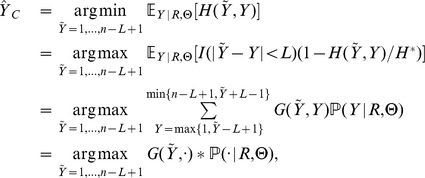
as required.

When contrasted to 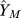 we can see the effect of having a higher resolution loss function: 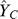 gathers probability support from nearby, relative to 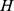, binding site configurations instead of just picking the most likely configuration. More specifically, for 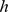 that corresponds to the overlap in binding sites, we have 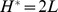, 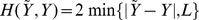, and so 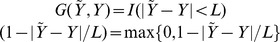, a “step pyramid” convolution filter that weights farther contributions less heavily. *From now on we will be adopting this loss/gain function.*

Other choices of 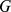 could be used, but they do not necessarily correspond to a generalized Hamming loss, and thus not to a centroid estimator (as defined here) either. The centroid estimator in [21], [22], for instance, adopts the thresholded gain 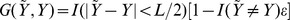, close to a “half plateau” filter, where 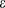 is an infinitesimal meant to break ties. Besides offering less resolution when comparing binding site configurations—binding sites are only compared for a “significant” overlap—this gain function does not result from a generalized Hamming loss since positional information is needed to assess if an overlap is significant or not. Finally, to get some insight into the new estimator, check the first example in the Results section.

### One Sequence, Multiple Binding Sites

We now allow for multiple binding sites by defining 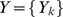 as the collection of *non-overlapping* binding sites 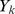. The likelihood is similar, but accounts for the multiple binding sites:
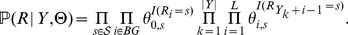
Given the “entropic” effect of possibly having many binding sites, we need to adopt a better prior for 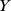 that takes into account the number of possible configurations for the binding sites. So, instead of naively electing 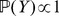, we explore a hierarchical structure: if 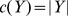, the number of binding sites in 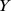, we note that 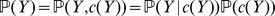, then first set 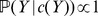
—binding site configurations are equally likely given the number of binding sites—and next define 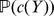.

In what follows we settle on a prior distribution for 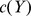 that is based on a Markov chain with two states, background and motif, where the probability of transitioning to background, either from background or motif, and of starting at background is 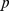; this approach results in
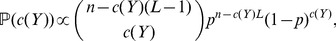
(3)since there needs to be 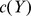 transitions to the motif state. This prior structure offers a good degree of flexibility through 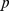: we can further set a hyperprior distribution on 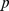, or specify it directly based on the expected number 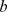 of binding sites in the sequence; if 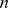 is large compared to 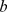, as usual, then 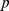 should be close to one, 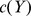 is approximately Poisson with mean 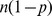 and thus 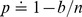 becomes a good candidate.

Going back to our inferential goal we note that, in contrast to the one binding site case from last section, posterior inference is more difficult since comparing configurations with different number of binding sites is not amenable to a systematic approach. Our first approximation is to consider local estimators for each group of configurations with a fixed number of binding sites and then appeal to a triangle inequality:
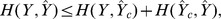
where 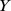 is a configuration with 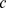 binding sites, 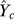 is the constrained estimator for all configurations with 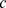 binding sites, and 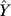 is the (overall) centroid estimator. Let 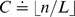 be the maximum number of binding sites in 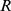, and recall that for the centroid estimator we wish to find 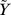 that minimizes
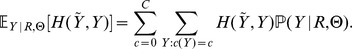
Using the triangle inequality for each group we then have
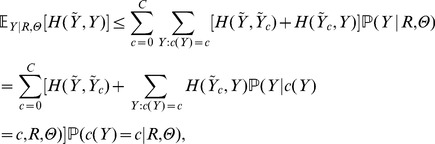
(4)where 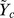 is an arbitrary point in 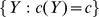. Our task is now to find an estimator—let us still call it centroid—that minimizes the right-hand bound in Equation 4 above. This goal suggests a two-step strategy:

1. For each number of binding sites, 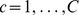, find the *local* centroids
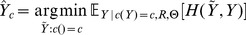
(5) as the 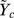 in Equation 4.
2. Find the *global* centroid given the local centroids 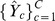,
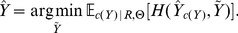
(6)

We note that this strategy does not guarantee that the bound is minimized; the main goal here is computational convenience. Let us tackle each step of this heuristic next. To this end we need 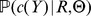 and marginal posteriors 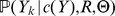; obtaining these posterior probabilities is a standard procedure, but we provide details on how they can be computed in File S1 for completeness.

### Local Centroids

Even when the number of binding sites is fixed, minimizing the conditional posterior expectation of 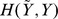 can be challenging: we would still have to consider for each candidate configuration 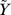 the posterior probability of configurations with all binding sites to the left of the first binding site in 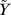, in-between binding sites in 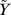, and so on. We adopt another approximation and decide to minimize a *paired* Hamming loss 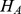 where binding site positions are matched according to their order:
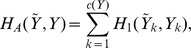
where 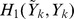 is Hamming loss when comparing sequences with only one binding site at 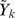 and 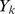, respectively, that is, 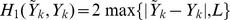. From the definition we have that 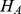 upper bounds 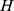: 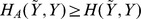. As a bad approximation example, if 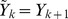 for 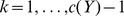 then 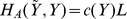, since each pair of binding sites 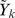 and 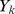 does not overlap, while 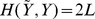 since only 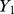 and 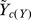 are in disagreement with background.

The next result adapts Theorem 1 to yield the paired local centroids.

**Lemma 2** *If* 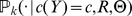 *is the marginal conditional posterior on* 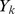 *then the paired local centroids are*

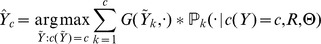



*Proof.* In the same spirit of Theorem 1, we use the conditional estimator in Equation 5 with the paired loss 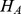:
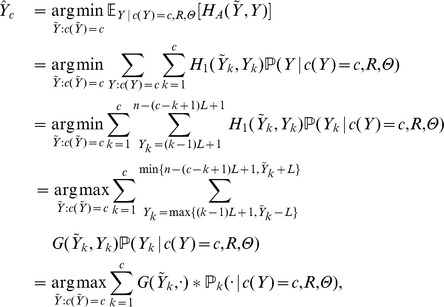
and the result follows.

We can spot in Lemma 2 the familiar convolutions, but now with the marginal posteriors 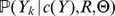 and in a more restricted range. We have a nice characterization, but we still have to optimize a sum to obtain the local centroids; to this end we explore the same recursive structure that allows us to compute forward and backward sums (see File S1 for details.) Let us define 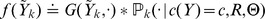 as the convolution against the marginal posterior on 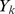; then we should have
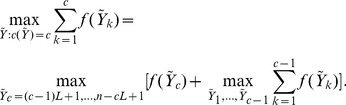
(7)This important observation allows us to obtain 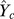 using the dynamic programming approach listed in Algorithm 1, as Theorem 3 formalizes.

**Algorithm 1** Find 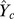 using dynamic programming.

*Construct partial maxima and backtrack pointers:*

Step 1. Set 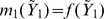 for 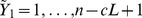.

Step 2. For 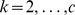 and 
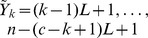
 do: set backtrack pointers
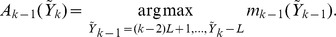
and set partial maximum sum 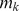 as
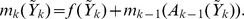



*Reconstruct centroid* 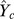 *using backtrack pointers:*

Step 3. Set last binding site position:
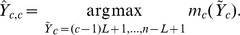
Note that, by construction, 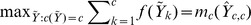.

Step 4. For 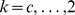 do: recover the remainder of 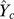 by setting 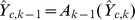.

**Theorem 3** *Algorithm 1 correctly identifies the paired local centroids*

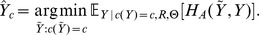



*Proof.* From Lemma 2 we know that 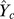 is the argument of 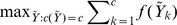. The key device in Algorithm 1 is to exploit the recursion in Equation 7 to define 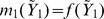 and
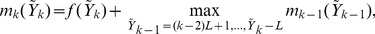
(8)for 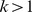, to store partial sum maxima. Now it follows that
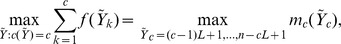
and so Step 3 must be correct. The correctness of Step 4 relies on the right specification of 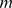 in Steps 1 and 2; but these steps are a straightforward application of Equation 7 using the definition of 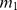 and a formulation of Equation 8 based on the backtrack pointers 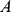, and so the algorithm is correct.

We note that the paired local centroids minimize an expected posterior upper bound 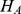 on the loss 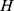, and so the actual local centroid might not be attained. We expect, however, that for common cases in which the motif coverage 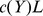 is much smaller than 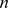 that the bound is tight since 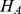 approximates 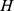 well and thus the two local centroids often coincide.

### Global Centroid

While the local centroids already convey information about the distribution of posterior mass in the space of binding site configurations, the end goal of the analysis is a point estimate that is, in itself, a good representative of the space. Following the strategy we outlined in the beginning of this section, we can further summarize the information in the local centroids by identifying a configuration 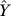 that minimizes the expected conditional Hamming loss, as in Equation 6. This approach, however, entails the same difficulties as defining the centroid based on all points in the space, and it is thus not treatable by a systematic approach—we are now just restricting the configurations to the local centroids.

The global centroid can be defined by direct enumeration of all possible configurations while keeping the minimizer of the expected conditional posterior loss, but this “brute-force” approach considers an exponential number of solutions. A simple heuristic is to restrict the global centroid to be one of the local centroids,
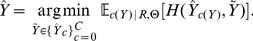
(9)Another alternative is to just take as global centroid the local centroid of the modal number of binding sites, 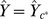, where 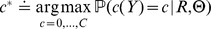. From now on we adopt the global centroid in Equation 9 for simplicity and, again, computational expediency.

### Constrained Global Centroid

A *constrained*, on the number of binding sites, global centroid might be more computationally feasible since we are restricting the space of available configurations. For instance, consider the 1-global centroid,
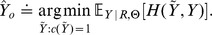
As when defining local centroids, we can approximate 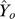 using a paired loss, and since
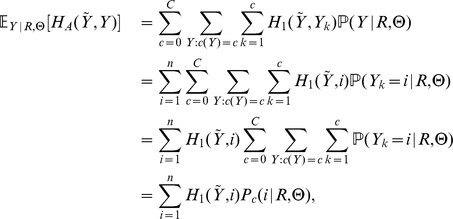
where
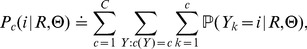
(10)we have that
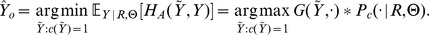
It is important to note that while the restriction of one binding site might seem artificial, the derivation of 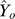 is helpful in recognizing sequence regions that are likely to host binding sites. In fact, since 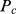 captures the posterior probability of having a binding site starting at each position, and considering the overlap gain 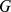, the convolution of 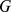 and 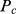 highlights positions that have higher posterior probability of being covered by a binding site.

### Multiple Sequences, Multiple Binding Sites per Sequence, Random Motif

We are now ready to address our model in broader generality: the dataset now comprises 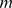 sequences, 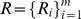, and thus binding site configurations are also indexed by sequence, 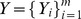. As before, we have that 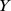 is independent of motif parameters 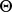, but we further assume that sequences and configurations are conditionally independent given 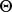:
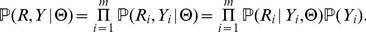
(11)


Given 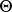 we would be able to apply the methods discussed this far to each sequence separately: compute forward and backward sums to obtain marginal posterior probabilities for each 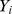 and then find local centroids and the 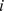-th global centroid. We will, however, assume that 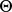 is random, say,
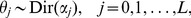
(12)independently with the usual conjugacy [14], and we thus wish to also conduct inference on background and motif compositions. This assumption, albeit more realistic, complicates matters, since the marginal unconditioned posterior distributions of 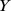 and 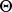 are not readily available; we are now required to estimate them before obtaining centroid estimates.

To obtain the centroids we follow the procedure described in the last section, but adopting Monte Carlo estimates of the marginal posterior distributions, for 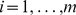,
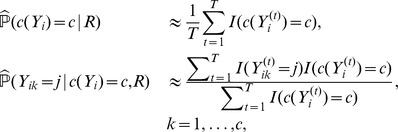
where 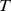 is the number of samples. In File S1 we present a Gibbs sampler [27], [28] that draws 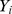 for each sequence given 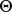 and then samples 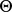 conditional on the binding site configurations 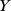, similar to the approach in [14].

## Results

### Illustrative Examples

#### Example 1: One Sequence, One Binding Site

Consider the following sequence of length 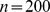 from the nucleotide alphabet 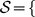
A, C, G, T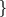,

 10 20 30 40 50

 | | | | |

**Figure 1:**
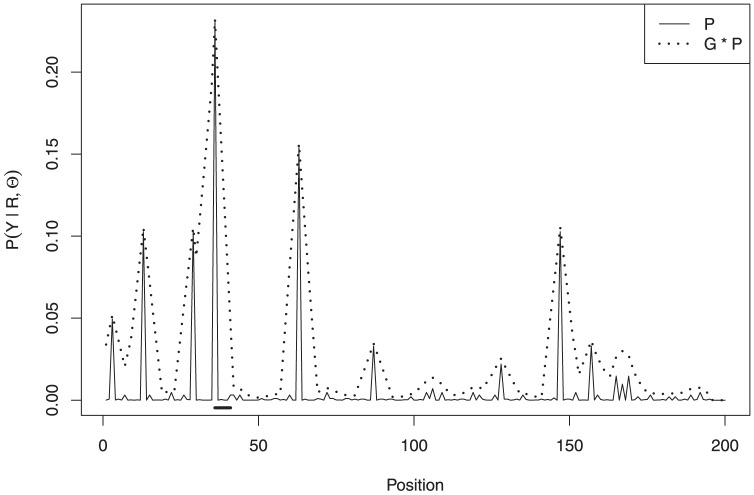
Conditional marginal probability distribution 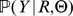 in solid line and convolution 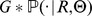 in dotted line. The black thick line close to the axis marks the binding site corresponding to the centroid 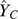.

GCCACTTTCGGGCCCGTGTCTAACGCACCACGGGCTACGTGACGGTGTGG CTCTATACTGACGACGTGAACCAAGCTTTACTGAAGGACTTGCTGTTCCC CGACCCATTTCCTGCCAGAACCTCTGACCAGTGTCTAGGGCTATCGCCCG TGATGTCTCATGGCGACGCGCGAGGCGGTTGCTCGCCTCACTCCGTTCTG

and a motif of length 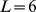 with parameters 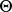 given by Table 1. Figure 1 shows the conditional marginal posterior 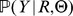 and the convolution 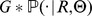 used to obtain the centroid 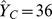, binding at the subsequence TACGTG, close to the consensual motif. Note that since 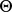 is very informative the posterior profile has clear peaks and in this case 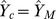, the two estimators coincide.

**Table 1:** Background and motif compositions.

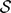	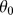	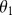	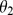	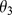	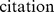	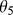	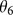
A	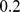	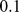	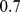	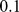	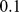	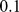	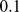
C	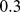	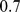	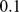	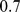	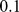	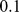	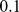
G	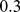	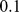	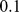	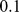	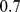	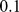	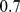
T	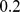	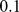	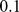	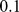	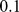	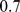	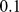

Background is assumed to be CG-rich, while the motif represents a canonical palindromic E-box, CACGTG [34].

#### Example 2: One Sequence, Multiple Binding Sites

We revisit the same sequence from Example 1, but now allow for at most 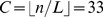 binding sites, and adopt the prior given in Equation 3 with 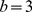 and thus 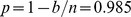. Using Algorithm 1 in File S1 we are able to compute the conditional marginal posteriors 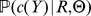 and 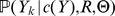 for 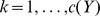. These posterior distributions yield the local centroids—according to Algorithm 1—and the global centroid from Equation 9. In Table 2 we list the marginal posterior 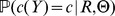 up to the smallest 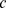 such that 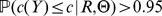, along with the local centroids; the global centroid 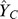 is highlighted. Interestingly, the global centroid coincides with the local centroid from the modal number of binding sites.

**Table 2:** Centroids and marginal posterior distribution of number of binding sites.

*c*	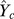	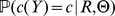	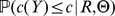
0	–	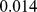	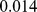
1	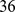	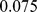	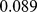
2	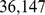	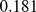	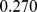
**3**	**13,36,147**	**0.254**	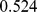
4	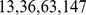	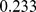	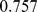
5	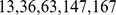	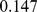	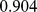
6	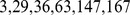	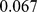	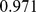

The global centroid and the modal number of binding sites are highlighted in bold.

**Figure 2:**
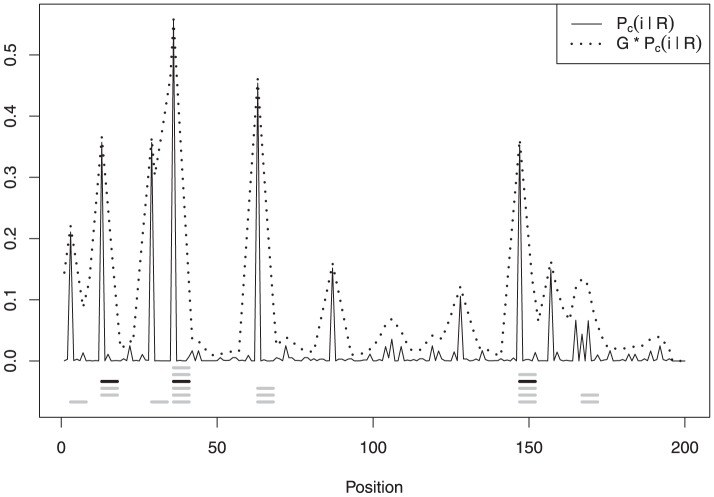
Posterior binding site coverage 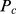 in solid line and convolution 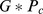 in dotted line. Local centroids are listed below in gray; the global centroid is in black.

In Figure 2 we display the posterior probabilities of binding site coverage 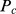 from Equation 10, along with the convolutions that are needed to define the 1-global centroid 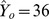. As can be seen, position 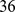 has a lot of support, being present in all the local centroids listed in Table 2; in fact, the probability of a binding site starting at position 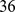 is greater than 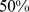.

**Figure 3:**
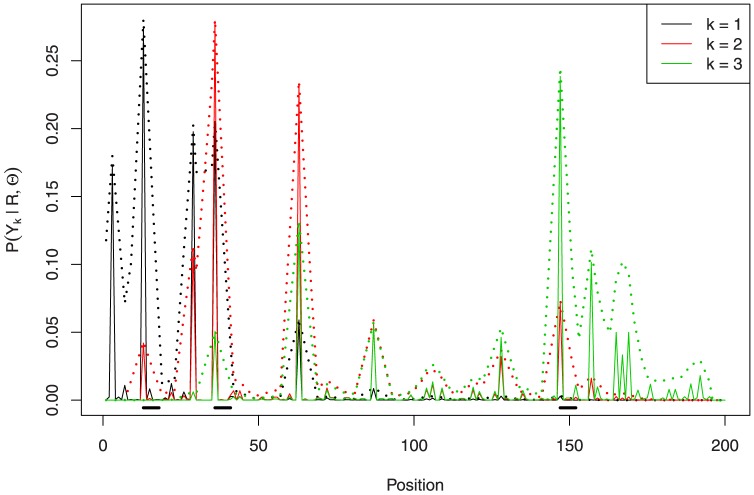
Marginal posterior distributions 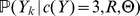 in solid line and convolutions 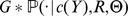 in dotted line. The local centroid is displayed at the bottom.

While 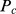 can provide us guidance for which positions are likely to start a binding site, using 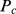 to define local centroids can be misleading. For instance, we could expect that the local centroid with three binding sites—the modal number of binding sites—would be, following a decreasing order on 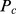, 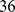, 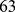, and 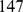. However, if we examine the marginal posteriors 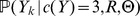 in Figure 3 we realize that position 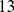 is favored over position 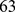 because, if 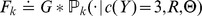, 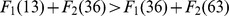.

#### Example 3: Multiple Sequences, Multiple Binding Sites per Sequence

For the random motif version of the last example we simulate 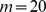 sequences of same length 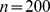 using 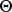 from Table 1 and the prior for 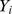, 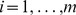, from Equation 3 with 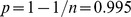. We continue focusing on the inference of binding site configurations in the same sequence from previous examples, which is the first sequence in the simulated dataset. We assume a non-informative prior on 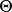 by setting 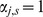 for 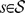 and 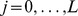; the prior on each sequence 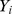 is the same prior from Example 2 with 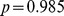. Algorithm 2 in File S1 is run for 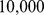 iterations to guarantee convergence (diagnostics not shown.)

**Figure 4:**
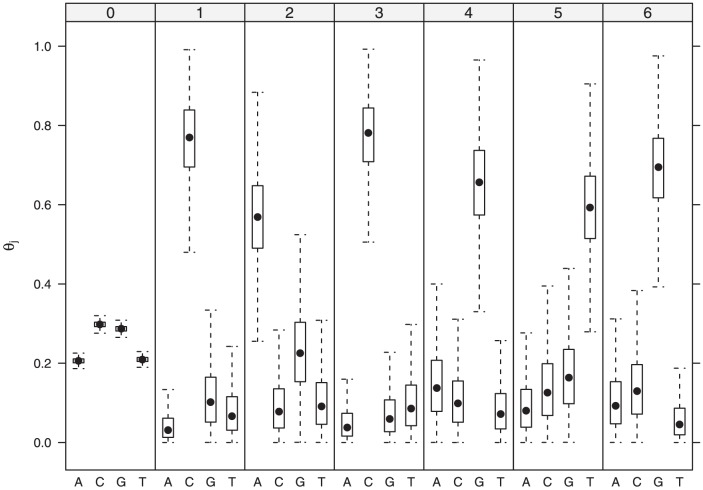
Boxplots of MCMC samples for 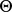. (Outliers are not shown).

The marginal posterior distribution of 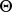 can be assessed in Figure 4. Since most positions in the sequences are background sequences 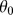 has very small posterior variances. Also note that the canonical palindromic E-box motif, with consensus CACGTG, is recovered. The procedure is now similar to what we presented in Example 2; the main difference is that the marginal posterior distributions are estimated from Markov chain Monte Carlo (MCMC) samples obtained as shown in File S1. Table 3 lists the estimated marginal posterior distribution of the number of binding sites, the local and global centroids. The global centroid does not coincide with the local centroid for the modal number of binding sites. Moreover, the local centroids here are different from the (conditional) local centroids in the last example, most likely due to the randomness of 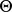 being taken into account.

**Table 3:** Centroids and estimated marginal posterior distribution of number of binding sites.

*c*	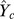	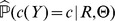	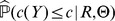
0	–	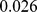	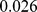
1	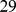	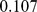	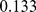
**2**	**29,167**	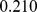	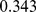
3	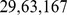	**0.274**	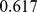
4	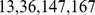	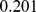	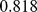
5	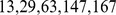	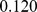	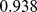
6	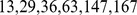	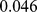	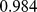

The global centroid and the modal number of binding sites are highlighted in bold.

**Figure 5:**
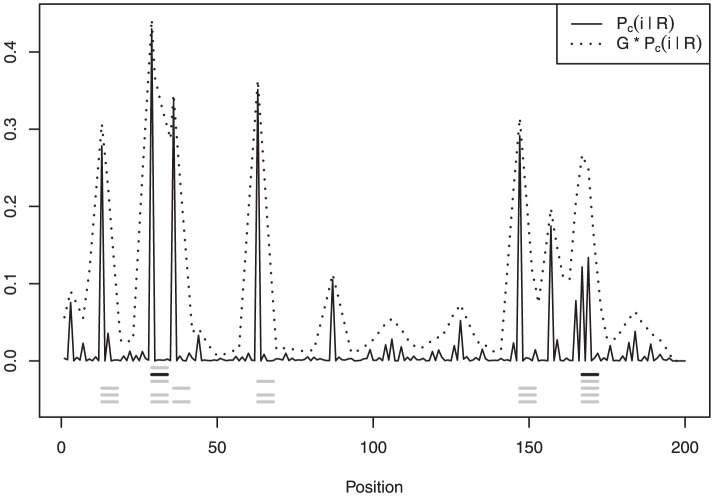
Estimated posterior binding site coverage 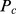 in solid line and convolution 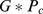 in dotted line. Local centroids are listed below in gray; the global centroid is in black.

Figure 5 displays the estimated 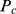, 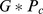, and the centroids. We see that compared to the second example some posterior mass has shifted to positions 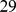 and to the group of positions 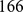, 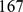, and 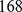. Here we clearly see the advantage of a centroid estimator: 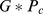, and later 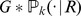, gathers evidence of motif binding from nearby positions, yielding a better summary—according to our choice of loss function—of the distribution of posterior mass.

**Figure 6:**
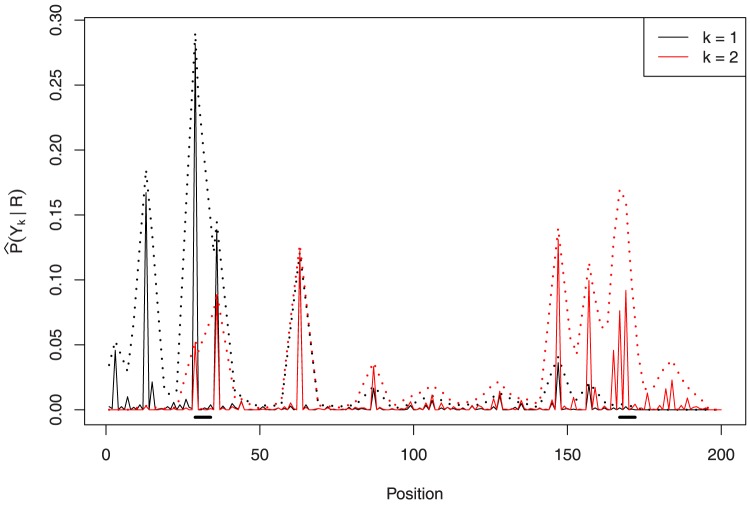
Estimated marginal posterior distributions 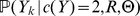 in solid line and convolutions 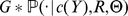 in dotted line. The local centroid is displayed at the bottom.

The selection of position 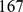 in the second local centroid 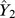 might seem puzzling since the peaks at positions 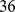, 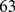, and 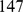 hold higher coverage probabilities. Checking 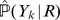 in Figure 6 helps dismiss any doubts: most of the support for these positions come from configurations with higher number of binding sites, as evidenced by the respective local centroids, but these configurations hold relatively low posterior mass. When 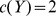, the prior on 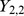 assigns more posterior probability to higher positions, close to the end of the sequence, simply because there are more configurations for 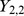 on these positions. It is also important to notice that while none of the positions in the cluster 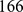
–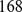 has higher marginal posterior mass than positions 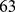 and 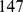, the convolution 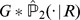 is maximized at position 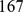, that is, the cluster when taken together has more support from the data, as weighted by 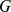.

### Case Study

We end this section with an example from the real-world dataset in [5], sequence set yst02r. The dataset contains 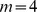 sequences each with 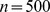 letters. We set 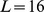 and adopt a non-informative prior on 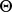, as in the previous example, and the prior on each 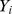, for the 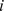-th sequence, from Equation 3 with 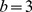 per thousand positions, so 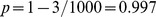. As in the previous example, 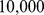 iterations suffice to reach convergence.

**Figure 7:**
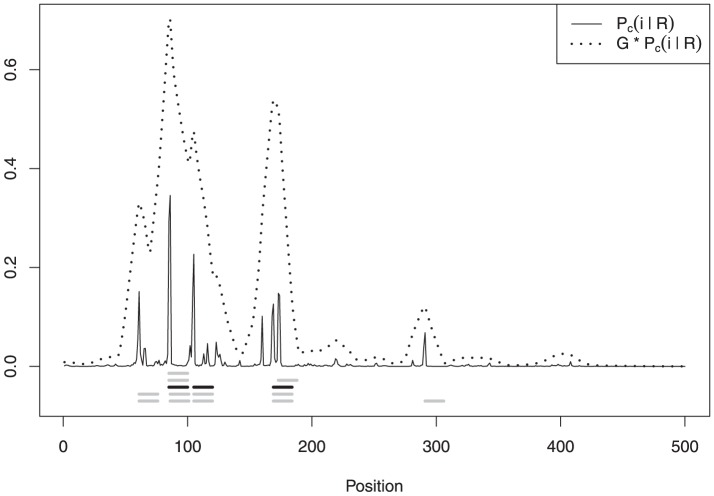
Estimated posterior binding site coverage 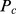 and convolution 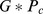 for real-world dataset, second sequence. Binding site coverage 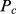 in solid line and convolution 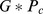 in dotted line. Local centroids are listed below in gray; the global centroid is in black.

Let us focus on the second sequence. Figure 7 pictures the binding site coverage probabilities, along with the local centroids. The global centroid 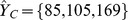 contains three binding sites, and it is also the local centroid for the modal number of binding sites, with 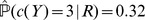. Since most of the posterior mass in concentrated in configurations with 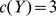, the posterior profiles 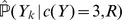 are similar to 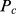 and are thus omitted.

**Figure 8:**
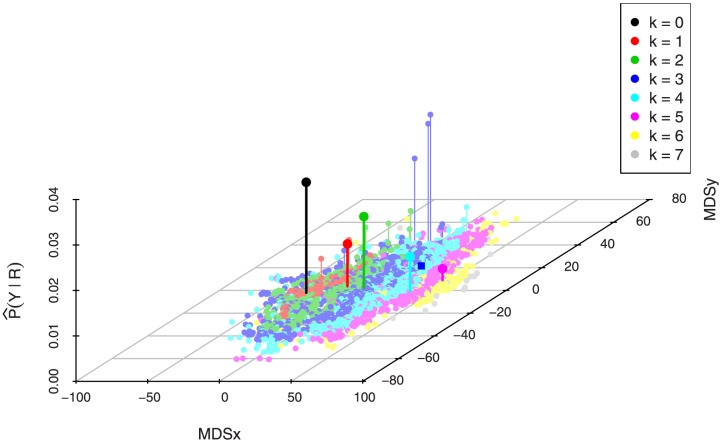
Estimated posterior distribution of configurations 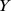 based on MCMC samples and projected using multidimensional scaling. The colors code configurations with different number of binding sites. Bold points mark local centroids, while a square (bold) point highlights the global centroid.

From the MCMC samples we can produce the MAP estimate 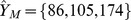 as the configuration with highest frequency among the samples: 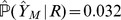. In fact, we can estimate the posterior probability of each sampled binding site configuration and then, using classic multidimensional scaling [29], visualize the estimated posterior distribution in Figure 8. It is interesting to note that the null configuration—that is, without binding sites—is also very likely with posterior probability 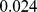. In contrast, the global centroid has very small posterior probability, close to 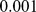; it sits, however, closer to configurations with high posterior mass, including the local centroids with one, two, and four binding sites.

**Figure 9:**
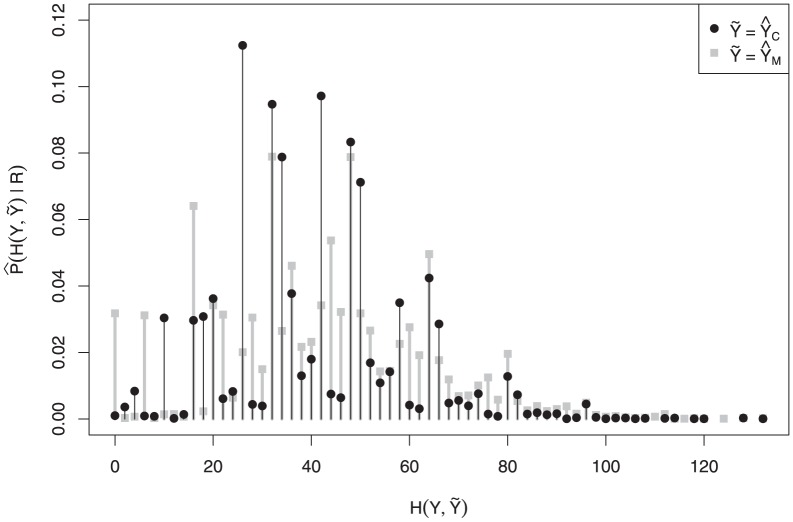
Estimated posterior distribution of loss function centered at 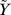 for the MAP (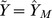) and centroid (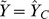) estimates.

To better assess how the centroid estimator is closer to a mean than a mode estimator, we plot the estimated posterior distribution of the generalized loss function 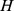 centered at both 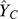 and 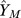 in Figure 9. Since 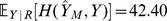 and 
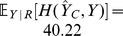
, we see that the binding sites in the centroid configuration are, on average, overlapping two extra positions with the binding sites in all the configurations when compared to the MAP estimate's binding sites. Both estimates are fairly similar, but the centroid reminds us that placing the third binding site at position 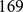, instead of 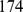, yields an unlikely configuration, but with a higher chance of overlapping with binding sites in positions 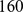
–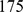 that have high posterior probability. In the context of Figures 8 and 9, the centroid places itself between two clusters that concentrate posterior mass: one with configurations 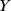 such that 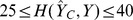 and another with configurations further away, satisfying 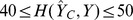.

## Discussion

In this paper we have presented a Bayesian approach, similar to the Gibbs motif sampler in [12], [14], that jointly models motif and background compositions and binding site locations in a set of sequences. More importantly, we discuss and formalize an inferential procedure based on the centroid estimator proposed by Carvalho and Lawrence [20]. As in any Bayesian analysis, we wish to evaluate features of interest in a model based on their posterior distribution; however, if we are required to pick a representative configuration, a point in the parameter space, then a principled approach is to elect a loss function and conduct formal statistical decision analysis. In this sense, by exploring a more refined loss function that depends on position-wise comparisons between sequence states—background or motif positions—we are able to identify a better representative of the posterior space of binding site configurations. Perhaps more importantly, this loss function is meaningful to investigators since is commonly adopted as a metric to measure binding site level accuracy [4], [5], [25], and so the centroid estimator should be preferred over MAP estimation in principle. Moreover, as pointed out in [20], the centroid estimator better accounts for the distribution of posterior mass; it is more similar to a median than to a mode, and can thus offer better predictive resolution than the MAP estimator [18]. When applied to motif discovery, the centroid estimator captures information in the vicinity of binding site positions through a convolution in marginal posterior distributions of binding sites.

Given the combinatorial number of possible configurations in the parameter space it is not straightforward to identify the centroid estimate through enumeration or even a systematic approach. Yet, we devise an approximative scheme that efficiently optimizes an upper bound on the posterior expected loss and thus provides a related centroid. Despite its heuristic nature, the proposed method has another advantage besides computational convenience: it allows for an informative depiction of the posterior distribution on binding site configurations. First, when defining the local centroids, we are able to assess the contributions from each binding site through their marginal posterior distributions conditional on the number of binding sites, and, in particular, through the convolution of these marginal profiles with the gain filter; secondly, when finding the global centroid we explore the marginal posterior distribution on the number of binding sites. Moreover, other representations might be helpful in understanding the distribution of posterior mass, as in the use of 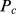 (in Equation 10) to pinpoint the 1-global centroid and measure the overall support of the configurations to a binding site at some specific position in the sequence. These comments are in the spirit of an estimator being also a communicator of the posterior space and the particular choice of prior distribution (see, e.g., Section 4.10 in [24].)

It is important to note that even when the model is accurate, poor inference might fail in recovering relevant features of the space. In Example 2, the MAP estimate is the null configuration, while the centroid indicates three binding sites that represent a group of configurations that jointly pool significant posterior mass. It is also common that the posterior distribution is too complex to be reasonably captured by a single representative; in this case the expected posterior loss could also be used to partition the space and further define additional representatives as conditional estimates on each subspace. This is a direction of work that warrants interest and that we intend to follow next.

Product multinomial and product Dirichlet models are justified as a good working, first approximation based on position independence. There are many extensions to this model that consider DNA strand complementarity [30], a more informative Markov structure for the background composition [31], and an explicit representation of the number of binding sites per sequence [32]. While we adopted a simple hierarchical model to guide the discussion, the proposed methodology is actually broader and the centroid estimators can be obtained from any Bayesian procedure that reports marginal posterior probabilities 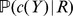 and 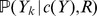, 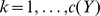, for sequence 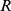 and binding site configuration 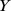.

Further improvements can be obtained by specifying a more complex model that accounts, for example, for higher order Markov chains with more states for the background, as in [30], [31], phylogenetic profiles [22], structural information [33], a variable motif length, or dependency among motif positions. As pointed out by Hu, Li, and Kihara [4], motif discovery using sequence only is well known for low signal-to-noise ratio; future extensions would also incorporate other data sources, such as gene expression or ChIP-Seq data, to increase the signal-to-noise ratio. In addition, for future work we intend to extend the model to account for multiple motifs, either from multiple TFs or from a single TF with alternative motifs. While the problem then becomes computationally more challenging, we expect that recursions and estimators similar to the ones discussed here will follow from extra bookkeeping on which motifs are bound at each binding site.

## Supporting Information

File S1
**Derivation of conditional and marginal posterior probabilities for** 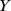 **and** 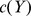 **and Gibbs sampler for the posterior joint on** 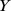 **and** 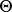 **.** Derivation of conditional posterior probabilities 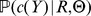 and marginal posterior probabilities 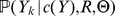, along with a routine to compute them in Algorithm 1. A Gibbs sampler to iteratively sample 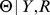 and 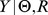 is given in Algorithm 2.(PDF)
