# Supplementary material for: Bayesian Centroid Estimation for Motif Discovery
Source: PLoS One. 2013 Dec 6;8(12):e80511. doi: 10.1371/journal.pone.0080511 (PMC3855595; doi:10.1371/journal.pone.0080511)
Supplement: File S1 — Derivation of conditional and marginal posterior probabilities for and and Gibbs sampler for the posterior joint on and . Derivation of conditional posterior probabilities and marginal posterior probabilities , along with a routine to compute them in Algorithm 1. A Gibbs sampler to iteratively sample and is given in Algorithm 2. (PDF) [file pone.0080511.s001.pdf]

Supporting Information S1 for "Bayesian Centroid Estimation for Motif Discovery"

Luis Carvalho*
Department of Mathematics and Statistics, Boston University, Boston, MA, USA
* E-mail: lecarval@math.bu.edu

1 Posteriors Conditional on Θ

In Methods we defined a hierarchical structure on Y and $c(Y)$ that lead us to a strategy for defining local and global centroid estimators. Here we outline how to compute the needed posteriors $\mathbb{P}(c(Y) \mid R, \Theta)$ and the marginal posteriors $\mathbb{P}(Y_k \mid c(Y), R, \Theta)$.

The posterior on Y is

$$\begin{aligned}\mathbb{P}(Y \mid R, \Theta) &= \frac{\mathbb{P}(R, Y \mid \Theta)}{\sum_{\widetilde{Y}} \mathbb{P}(R, \widetilde{Y} \mid \Theta)} \\ &= \underbrace{\frac{\mathbb{P}(R, Y \mid \Theta)}{\sum_{\widetilde{Y}: c(\widetilde{Y}) = c(Y)} \mathbb{P}(R, \widetilde{Y} \mid \Theta)}}_{\mathbb{P}(Y \mid c(Y), R, \Theta)} \cdot \underbrace{\frac{\sum_{\widetilde{Y}: c(\widetilde{Y}) = c(Y)} \mathbb{P}(R, \widetilde{Y} \mid \Theta)}{\sum_{c=0}^{C} \sum_{\widetilde{Y}: c(\widetilde{Y}) = c} \mathbb{P}(R, \widetilde{Y} \mid \Theta)}}_{\mathbb{P}(c(Y) \mid R, \Theta)}.\end{aligned}$$

By the structure of our prior it follows that

$$\begin{aligned}\mathbb{P}(Y \mid c(Y), R, \Theta) &= \frac{\mathbb{P}(R \mid Y, \Theta)\mathbb{P}(Y)}{\sum_{\widetilde{Y}: c(\widetilde{Y}) = c(Y)} \mathbb{P}(R \mid \widetilde{Y}, \Theta)\mathbb{P}(\widetilde{Y})} \\ &= \frac{\mathbb{P}(R \mid Y, \Theta)}{\sum_{\widetilde{Y}: c(\widetilde{Y}) = c(Y)} \mathbb{P}(R \mid \widetilde{Y}, \Theta)},\end{aligned} \tag{1}$$

and

$$\begin{aligned}\mathbb{P}(c(Y) \mid R, \Theta) &= \frac{\sum_{\widetilde{Y}: c(\widetilde{Y}) = c(Y)} \mathbb{P}(R \mid \widetilde{Y}, \Theta)\mathbb{P}(\widetilde{Y})}{\sum_{c=0}^{C} \sum_{\widetilde{Y}: c(\widetilde{Y}) = c} \mathbb{P}(R \mid \widetilde{Y}, \Theta)\mathbb{P}(\widetilde{Y})} \\ &= \frac{\sum_{\widetilde{Y}: c(\widetilde{Y}) = c(Y)} \mathbb{P}(R \mid \widetilde{Y}, \Theta)\mathbb{P}(\widetilde{Y} \mid c(\widetilde{Y}))\mathbb{P}(c(\widetilde{Y}))}{\sum_{c=0}^{C} \sum_{\widetilde{Y}: c(\widetilde{Y}) = c} \mathbb{P}(R \mid \widetilde{Y}, \Theta)\mathbb{P}(\widetilde{Y} \mid c(\widetilde{Y}))\mathbb{P}(c(\widetilde{Y}))}.\end{aligned} \tag{2}$$

We anticipate that this decomposition suggests a good approach to sampling from $\mathbb{P}(Y \mid R, \Theta)$: we can first sample $c(Y)$ according to $\mathbb{P}(c(Y) \mid R, \Theta)$ and then sample Y given the number of binding sites, according to $\mathbb{P}(Y \mid c(Y), R, \Theta)$.

1.1 Marginal Posterior on $c(Y)$

From Equations 1 and 2 we observe that we need to compute $\sum_{\widetilde{Y}: c(\widetilde{Y}) = c} \mathbb{P}(R \mid \widetilde{Y}, \Theta)$ up to a constant to find both conditional posteriors of $c(Y)$ and Y and thus the posterior $\mathbb{P}(Y \mid R, \Theta)$. Let us now denote by $R_{i:j}$ the subsequence of R from positions i to j and by $Y_{i:j}$ the binding sites in Y between i and j—that is, all Y_k such that $i \le Y_k \le j - L + 1$. If we then define *forward sums*

$$F_{c,j} \doteq \frac{\sum_{\widetilde{Y}_{1:j}: c(\widetilde{Y}_{1:j}) = c} \mathbb{P}(R_{1:j} \mid \widetilde{Y}_{1:j}, \Theta)}{\prod_{i=1}^{j} \prod_{s \in \mathcal{S}} \theta_{0,s}^{I(R_i = s)}} \tag{3}$$

we have that $\sum_{\widetilde{Y}:c(\widetilde{Y})=c} \mathbb{P}(R \,|\, \widetilde{Y}, \Theta) \propto F_{c,n}$. To further simplify the notation, let us define

$$\lambda(j;\Theta) = \prod_{i=1}^{L} \prod_{s\in\mathcal{S}} \left(\frac{\theta_{i,s}}{\theta_{0,s}}\right)^{I(R_{j-1+i}=s)},$$

the composition ratio between motif and background for a binding site starting at j.

The forward sums $F_{c,j}$ can be computed recursively,

$$F_{c,j} = F_{c,j-1} + F_{c-1,j-L}\lambda(j-L+1;\Theta), \tag{4}$$

by considering two options for the tail of the sequence: either having a background position—and hence the first summand above—or by having a binding site on the last L positions—and thus requiring the second summand.

Thus, we have

$$\mathbb{P}(c(Y) \,|\, R, \Theta) = \frac{F_{c(Y),n} \binom{n-c(Y)(L-1)}{c(Y)}^{-1} \mathbb{P}(c(Y))}{\sum_{c=0}^{C} F_{c,n} \binom{n-c(L-1)}{c}^{-1} \mathbb{P}(c(Y)=c)}, \tag{5}$$

which yields a straightforward way to sample the posterior $c(Y)$ conditional on Θ.

1.2 Marginal Posterior on Y_k Given $c(Y)$

To compute $\mathbb{P}(Y_k \,|\, c(Y), R, \Theta)$ we now need backward sums. We can define them analogously to the forward sums:

$$B_{c,j} \doteq \frac{\sum_{\widetilde{Y}_{j:n}:c(\widetilde{Y}_{j:n})=c} \mathbb{P}(R_{j:n} \,|\, \widetilde{Y}_{j:n}, \Theta)}{\prod_{i=j}^{n} \prod_{s\in\mathcal{S}} \theta_{0,s}^{I(R_i=s)}}, \tag{6}$$

and hence $\sum_{\widetilde{Y}:c(\widetilde{Y})=c} \mathbb{P}(R \,|\, \widetilde{Y}, \Theta) \propto B_{c,1}$, as expected. Moreover, by a similar argument to the previous subsection, we also have that the backward sums are recursive:

$$B_{c,j} = B_{c,j+1} + B_{c-1,j+L}\lambda(j;\Theta). \tag{7}$$

Having forward and backward sums enable us to readily compute the marginal posterior on Y_k conditional on $c(Y)$: since

$$\begin{aligned}
\mathbb{P}(Y_k \,|\, c(Y)=c, R, \Theta) &= \sum_{Y_1,\ldots,Y_{k-1},Y_{k+1},\ldots,Y_c} \mathbb{P}(Y \,|\, c(Y)=c, R, \Theta) \\
&= \sum_{Y_1,\ldots,Y_{k-1},Y_{k+1},\ldots,Y_c} \frac{\mathbb{P}(R \,|\, Y, \Theta)}{\sum_{\widetilde{Y}:c(\widetilde{Y})=c} \mathbb{P}(R \,|\, \widetilde{Y}, \Theta)},
\end{aligned}$$

and

$$\begin{aligned}
\sum_{Y_1,\ldots,Y_{k-1},Y_{k+1},\ldots,Y_c} \mathbb{P}(R \,|\, Y, \Theta) = &\sum_{Y_1,\ldots,Y_{k-1}} \mathbb{P}(R_{1:Y_k-1} \,|\, Y_{1:Y_k-1}, \Theta) \\
&\cdot \mathbb{P}(R_{Y_k:Y_k+L-1} \,|\, Y_{Y_k:Y_k+L-1}, \Theta) \cdot \sum_{Y_{k+1},\ldots,Y_c} \mathbb{P}(R_{Y_k+L:n} \,|\, Y_{Y_k+L:n}, \Theta),
\end{aligned}$$

and thus

$$\mathbb{P}(Y_k \,|\, c(Y)=c, R, \Theta) = \frac{F_{k-1,Y_k-1}\lambda(Y_k;\Theta)B_{c-k,Y_k+L}}{\sum_{\widetilde{Y}_k=(k-1)L}^{n-(c-k+1)L+1} F_{k-1,\widetilde{Y}_k-1}\lambda(\widetilde{Y}_k;\Theta)B_{c-k,\widetilde{Y}_k+L}}. \tag{8}$$

Note that

$$\frac{\sum_{\widetilde{Y}:c(\widetilde{Y})=c}\mathbb{P}(R\,|\,\widetilde{Y},\Theta)}{\prod_{i=1}^{n}\prod_{s\in\mathcal{S}}\theta_{0,s}^{I(R_i=s)}} = F_{c,n} = B_{c,1} = \sum_{\widetilde{Y}_k=(k-1)L}^{n-(c-k+1)L+1} F_{k-1,\widetilde{Y}_k-1}\lambda(\widetilde{Y}_k;\Theta)B_{c-k,\widetilde{Y}_k+L},$$

for $k=1,\ldots,c$.

We summarize the results of this appendix in Algorithm 1.

Algorithm 1 Computes $\mathbb{P}(c(Y)\,|\,R,\Theta)$ and $\mathbb{P}(Y_k\,|\,c(Y),R,\Theta)$ for $k=1,\ldots,c(Y)$.

Step 1. *(Initialize)* Set $F_{0,0}=B_{0,n+1}=F_{0,j}=B_{0,j}=1$ for $j=1,\ldots,n$; for $c=1,\ldots,C$, set $F_{c,j}=0$ when $j<cL$ and $B_{c,j}=0$ when $j>n-cL+1$.

Step 2. *(Compute forward sums)* For $c=1,\ldots,C$ and $j=cL+1,\ldots,n$ do: set $F_{c,j}$ as in Equation 4,

$$F_{c,j}=F_{c,j-1}+F_{c-1,j-L}\lambda(j-L+1;\Theta)$$

Step 3. *(Compute $\mathbb{P}(c(Y)\,|\,R,\Theta)$)* For $c=0,\ldots,C$ do: compute marginal posterior $c(Y)$ as in Equation 5,

$$\mathbb{P}(c(Y)=c\,|\,R,\Theta)=\frac{F_{c,n}\binom{n-c(L-1)}{c}^{-1}\mathbb{P}(c(Y)=c)}{\sum_{\widetilde{c}=0}^{C}F_{\widetilde{c},n}\binom{n-\widetilde{c}(L-1)}{\widetilde{c}}^{-1}\mathbb{P}(c(Y)=\widetilde{c})}$$

Step 4. *(Compute backward sums)* For $c=1,\ldots,C$ and $j=n-cL,\ldots,1$ do: set $B_{c,j}$ as in Equation 7,

$$B_{c,j}=B_{c,j+1}+B_{c-1,j+L}\lambda(j;\Theta)$$

Step 5. *(Compute $\mathbb{P}(Y_k\,|\,c(Y),R,\Theta)$)* For $c=1,\ldots,C$, $k=1,\ldots,c$, and $Y_k=(k-1)L+1,\ldots,n-(c-k+1)L+1$ do: compute marginal posterior Y_k given $c(Y)$ as in Equation 8,

$$\mathbb{P}(Y_k\,|\,c(Y)=c,R,\Theta)=F_{k-1,Y_k-1}\lambda(Y_k;\Theta)B_{c-k,Y_k+L}/F_{c,n}$$

2 Gibbs Sampler

2.1 Sampling Θ Given Y and R

Since the prior on Θ is conjugate, we should be able to sample Θ exactly from a Dirichlet distribution. From Equations (11) and (12) in the main text we have

$$\begin{aligned}\mathbb{P}(\theta_0\,|\,Y,R) &\propto \left[\prod_{i=1}^{m}\prod_{s\in\mathcal{S}}\prod_{j\in BG_i}\theta_{0,s}^{I(R_{ij}=s)}\right]\left[\prod_{s\in\mathcal{S}}\theta_{0,s}^{\alpha_{0,s}-1}\right]\\ &=\prod_{s\in\mathcal{S}}\theta_{0,s}^{\sum_{i=1}^{m}\sum_{j\in BG_i}I(R_{ij}=s)+\alpha_{0,s}-1},\end{aligned}$$

and so $\theta_0\,|\,Y,R\sim\mathrm{Dir}(N_0(Y,R)+\alpha_0)$, where $N_0(Y,R)=\{N_{0,s}\}_{s\in\mathcal{S}}$ and

$$N_{0,s}=\sum_{i=1}^{m}\sum_{j\in BG_i}I(R_{ij}=s)$$

is the number of background positions across all sequences that have symbol s. Similarly, for the j-th position in the motif,

$$\begin{aligned}\mathbb{P}(\theta_j \mid Y, R) &\propto \left[\prod_{i=1}^{m}\prod_{s\in\mathcal{S}}\prod_{k=1}^{|Y_i|}\theta_{j,s}^{I(R_{i,Y_{ik}+j-1}=s)}\right]\left[\prod_{s\in\mathcal{S}}\theta_{j,s}^{\alpha_{j,s}-1}\right]\\ &= \prod_{s\in\mathcal{S}}\theta_{0,s}^{\sum_{i=1}^{m}\sum_{j\in BG_i} I(R_{ij}=s)+\alpha_{0,s}-1},\end{aligned}$$

and thus $\theta_j \mid Y, R \sim \text{Dir}(N_j(Y, R) + \alpha_j)$, with $N_j(Y, R) = \{N_{j,s}\}_{s\in\mathcal{S}}$ and

$$N_{j,s} = \sum_{i=1}^{m}\sum_{k=1}^{|Y_i|} I(R_{i,Y_{ik}+j-1} = s)$$

is the number of motif j-th positions across all sequences and binding sites that have symbol s.

2.2 Sampling Y_i Given Θ and R

Each configuration Y_i for the i-th sequence is conditionally independent given Θ, so we can devise a sampling procedure that can be applied to each sequence in turn. To simplify the notation, let us drop the sequence index in what follows, that is, Y_i is Y, R_i is R, and so on. We will be following a similar approach to Sections 1.1 and 1.2, but instead of summing to obtain marginal distributions we will be sampling *exactly*.

To sample from the conditional posterior on Y, we first sample $c(Y) = c$ according to Equation 5 and then proceed to sample Y from its last, c-th binding site up to its first binding site. For this reason, this strategy is commonly referred to as "stochastic backtracking", since it can be regarded as a stochastic version of Step 4 in Algorithm 1. Sampling Y is similar to the predictive update step in [1], which, on its turn, is based on a stochastic variation of expectation-maximization where missing data is imputed [2]; however, here we exploit a hierarchical structure on $c(Y)$ and do not use the collapsing technique of Liu [3].

Exploiting the conditional independence of the sequence configurations and Equation 1 the last binding site can be sampled using

$$\begin{aligned}\mathbb{P}(Y_c \mid c(Y), R, \Theta) &= \frac{\sum_{Y_1,\ldots,Y_{c-1}} \mathbb{P}(R \mid Y, \Theta)}{\sum_{\widetilde{Y}_c}\sum_{\widetilde{Y}_1,\ldots,\widetilde{Y}_{c-1}} \mathbb{P}(R \mid \widetilde{Y}, \Theta)}\\ &= \frac{F_{c-1,Y_c-1}\lambda(Y_c; \Theta)}{\sum_{\widetilde{Y}_c=(c-1)L+1}^{n-L+1} F_{c-1,\widetilde{Y}_c-1}\lambda(\widetilde{Y}_c; \Theta)}.\end{aligned} \tag{9}$$

To sample the (intermediate) j-th binding site we use a similar expression:

$$\begin{aligned}\mathbb{P}(Y_j \mid Y_{j+1}, \ldots, Y_c, c(Y), R, \Theta) &= \frac{\mathbb{P}(Y_j, \ldots, Y_c, c(Y), R, \Theta)}{\sum_{\widetilde{Y}_j} \mathbb{P}(\widetilde{Y}_j, \ldots, \widetilde{Y}_c, c(Y), R, \Theta)}\\ &= \frac{\sum_{Y_1,\ldots,Y_{j-1}} \mathbb{P}(R \mid Y, \Theta)}{\sum_{\widetilde{Y}_j}\sum_{\widetilde{Y}_1,\ldots,\widetilde{Y}_{j-1}} \mathbb{P}(R \mid \widetilde{Y}, \Theta)}\\ &= \frac{F_{j-1,Y_j-1}\lambda(Y_j; \Theta)}{\sum_{\widetilde{Y}_j=(j-1)L+1}^{Y_{j+1}-L} F_{j-1,\widetilde{Y}_j-1}\lambda(\widetilde{Y}_j; \Theta)}.\end{aligned} \tag{10}$$

By making the convention that $Y_{c+1} = |R| + 1$ we can reduce Equation 9 to Equation 10. Moreover, note that Equation 10 implies that

$$\mathbb{P}(Y_j \,|\, Y_{j+1}, \ldots, Y_c, c(Y), R, \Theta) = \mathbb{P}(Y_j \,|\, Y_{j+1}, c(Y), R, \Theta),$$

as expected.

We summarize the whole procedure in Algorithm 2. Note how Steps 1.1 to 1.3 are analogous to Steps 1 to 3 in Algorithm 1, and how Step 1.4 is an stochastic version of Step 4 in Algorithm 1: as previously stated, we are now sampling backwards instead of summing backwards.

Algorithm 2 Gibbs sampler for $\mathbb{P}(Y, \Theta \,|\, R)$.

Set $\Theta^{(0)}$ *arbitrarily. For* $t = 1, \ldots$ *(until convergence) do:*

Step 1. *(Sample* $Y \,|\, \Theta, R$*)* For each sequence $i = 1, \ldots, m$, do: let $n = |R_i|$, $C = \lfloor n/L \rfloor$ and sample $Y_i \,|\, R_i, \Theta$.

Step 1.1. *(Initialize)* Set $F_{0,j} = 1$ for $j = 0, 1, \ldots, n$ and for $c = 1, \ldots, C$ set $F_{c,j} = 0$ when $j < cL$.

Step 1.2. *(Compute forward sums)* For $c = 1, \ldots, C$ and $j = cL + 1, \ldots, n$ do: set $F_{c,j}$ as in Equation 4,

$$F_{c,j} = F_{c,j-1} + F_{c-1,j-L}\lambda_i(j - L + 1; \Theta^{(t-1)}),$$

where λ_i uses R_i.

Step 1.3. *(Sample* $c(Y_i^{(t)}) \,|\, R_i, \Theta^{(t-1)}$*)* For $c = 0, \ldots, C$ do: compute marginal posterior $c(Y_i)$ as in Equation 5 when applied to the i-th sequence,

$$\mathbb{P}(c(Y_i) = c \,|\, R_i, \Theta^{(t-1)}) = \frac{F_{c,n}\binom{n-c(L-1)}{c}^{-1}\mathbb{P}(c(Y_i) = c)}{\sum_{\widetilde{c}=0}^{C} F_{\widetilde{c},n}\binom{n-\widetilde{c}(L-1)}{\widetilde{c}}^{-1}\mathbb{P}(c(Y_i) = \widetilde{c})}$$

and sample $c^{(t)} \doteq c(Y_i^{(t)})$ according to $\mathbb{P}(c(Y_i) = c \,|\, R_i, \Theta^{(t-1)})$.

Step 1.4. *(Sample* $Y_i^{(t)} \,|\, c(Y_i^{(t)}) = c^{(t)}, R_i, \Theta^{(t-1)}$*)* For $k = c^{(t)}, \ldots, 1$ do: sample $Y_{ik}^{(t)}$ proportional to $F_{k-1,Y_{ik}^{(t)}-1}\lambda_i(Y_{ik}^{(t)}; \Theta^{(t-1)})$ as in Equation 10,

$$\mathbb{P}(Y_{ik}^{(t)} \,|\, Y_{i,k+1}^{(t)}, c(Y_i^{(t)}) = c^{(t)}, R_i, \Theta^{(t-1)}) = \\ = \frac{F_{k-1,Y_{ik}^{(t)}-1}\lambda_i(Y_{ik}^{(t)}; \Theta^{(t-1)})}{\sum_{\widetilde{Y}_k=(k-1)L+1}^{Y_{i,k+1}^{(t)}-L} F_{k-1,\widetilde{Y}_k-1}\lambda_i(\widetilde{Y}_k; \Theta^{(t-1)})}$$

Step 2. *(Sample* $\Theta \,|\, Y, R$*)* For $j = 0, \ldots, L$ compute $N_j(Y^{(t)}, R)$ and then sample $\theta_j^{(t)} \,|\, Y^{(t)}, R \sim \mathrm{Dir}(N_j(Y^{(t)}, R) + \alpha_j)$.

References

1. Liu J, Neuwald A, Lawrence C (1995) Bayesian models for multiple local sequence alignment and Gibbs sampling strategies. Journal of the American Statistical Association 90: 1156–1170.

2. Tanner M, Wong W (1987) The calculation of posterior distributions by data augmentation. Journal of the American Statistical Association 82: 528–540.

3. Liu J (1994) The collapsed Gibbs sampler in Bayesian computations with applications to a gene regulation problem. Journal of the American Statistical Association 89: 958–966.
